# Beyond the cochlea: exploring the multifaceted nature of hearing loss in primary mitochondrial diseases

**DOI:** 10.1093/braincomms/fcae374

**Published:** 2024-10-24

**Authors:** Nehzat Koohi, Sarah Holmes, Amanda Male, Doris-Eva Bamiou, Magdalena M Dudziec, Gita M Ramdharry, Chiara Pizzamiglio, Michael G Hanna, Robert D S Pitceathly, Diego Kaski

**Affiliations:** Department of Clinical and Movement Neurosciences, University College London Queen Square Institute of Neurology, London WC1N 3BG, UK; The Ear Institute, University College London, London WC1X 8EE, UK; NHS Highly Specialised Service for Rare Mitochondrial Disorders, Queen Square Centre for Neuromuscular Diseases, The National Hospital for Neurology and Neurosurgery, London WC1N 3BG, UK; Queen Square Centre for Neuromuscular Diseases, The National Hospital for Neurology and Neurosurgery, London WC1N 3BG, UK; Department of Clinical and Movement Neurosciences, University College London Queen Square Institute of Neurology, London WC1N 3BG, UK; The Ear Institute, University College London, London WC1X 8EE, UK; National Institute for Health Research, University College London Hospitals Biomedical Research Centre (Deafness and Hearing Problems Theme), London WC1X 8EE, UK; Queen Square Centre for Neuromuscular Diseases, The National Hospital for Neurology and Neurosurgery, London WC1N 3BG, UK; Department of Neuromuscular Diseases, University College London Queen Square Institute of Neurology, London WC1N 3BG, UK; Queen Square Centre for Neuromuscular Diseases, The National Hospital for Neurology and Neurosurgery, London WC1N 3BG, UK; Department of Neuromuscular Diseases, University College London Queen Square Institute of Neurology, London WC1N 3BG, UK; NHS Highly Specialised Service for Rare Mitochondrial Disorders, Queen Square Centre for Neuromuscular Diseases, The National Hospital for Neurology and Neurosurgery, London WC1N 3BG, UK; Department of Neuromuscular Diseases, University College London Queen Square Institute of Neurology, London WC1N 3BG, UK; NHS Highly Specialised Service for Rare Mitochondrial Disorders, Queen Square Centre for Neuromuscular Diseases, The National Hospital for Neurology and Neurosurgery, London WC1N 3BG, UK; Department of Neuromuscular Diseases, University College London Queen Square Institute of Neurology, London WC1N 3BG, UK; NHS Highly Specialised Service for Rare Mitochondrial Disorders, Queen Square Centre for Neuromuscular Diseases, The National Hospital for Neurology and Neurosurgery, London WC1N 3BG, UK; Department of Neuromuscular Diseases, University College London Queen Square Institute of Neurology, London WC1N 3BG, UK; Department of Clinical and Movement Neurosciences, University College London Queen Square Institute of Neurology, London WC1N 3BG, UK; The Ear Institute, University College London, London WC1X 8EE, UK

**Keywords:** hearing loss, mitochondrial disease, auditory brainstem responses, auditory processing, spatial processing disorder

## Abstract

Primary mitochondrial diseases, with diverse systemic manifestations, often present with auditory impairments due to mitochondrial dysfunction. This study provides an in-depth exploration of auditory deficits in primary mitochondrial diseases, highlighting the impact of various pathogenic variants on both cochlea and neural/central auditory functions. An observational study involving 72 adults with primary mitochondrial diseases was conducted. Participants underwent extensive audiological evaluations including pure-tone audiometry, tympanometry, acoustic reflex thresholds, quick speech-in-noise test, listening in spatialized noise-sentences test, auditory-evoked brainstem responses and distortion product otoacoustic emissions. Multivariate analysis of covariance and logistic regression analyses assessed the influence of various pathogenic DNA variants, accounting for age, cognitive status via the Montreal Cognitive Assessment and disease severity through the Newcastle Mitochondrial Disease Adult Scale. Participants with the pathogenic m.3243A>G/T variants (m.3243A>G *n* = 40; m.3243A>T *n* = 1) exhibited significant elevations in pure-tone audiometry thresholds, especially at high frequencies, suggesting cochlea involvement. Notably, the listening in spatialized noise-sentences test showed significant spatial processing deficits in the m.3243A>G/T group, possibly indicating a unique mutation-specific impact on central auditory processing. Auditory-evoked brainstem response results highlighted a higher likelihood of auditory brainstem response abnormalities in this group, further substantiating neural/central auditory pathway involvement. This study emphasizes the heterogeneous nature of hearing impairment in primary mitochondrial diseases, with a genotype–phenotype correlation, particularly in the m.3243A>G/T group. These insights advocate for personalized, genotype-specific auditory assessments and targeted management strategies. Conventional hearing aids and cochlear implants are ineffective for those with central auditory dysfunctions related to mitochondrial mutations. There is an urgent need for innovative rehabilitation strategies catering for both cochlear and neural/central auditory pathways.

## Teaser

Koohi *et al*. report that primary mitochondrial diseases often result in auditory impairments involving both cochlear and central auditory functions. Their study highlights genotype–phenotype correlations, particularly in the m.3243A>G/T-related primary mitochondrial diseases group, and emphasizes the need for personalized, genotype-specific auditory assessments and innovative rehabilitation strategies. Created in BioRender. Koohi, N. (2024) https://BioRender.com/y49r260

## Graphical Abstract

**Graphical Abstract:**
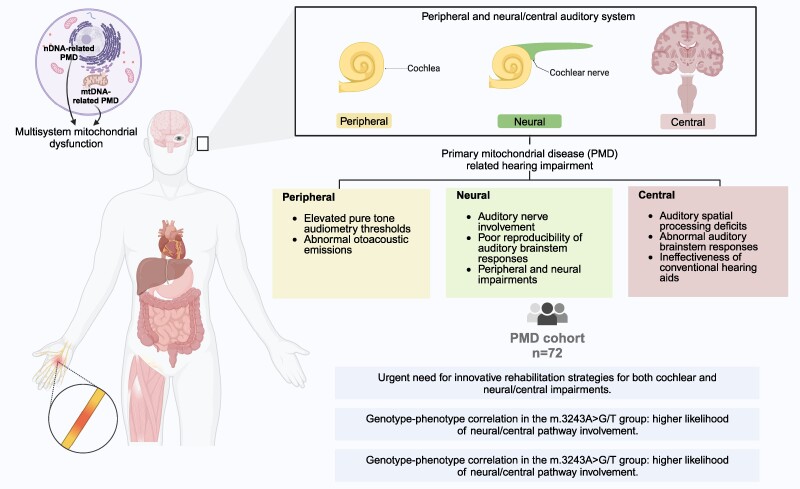

**See Di Stadio, M. Frohman, Messineo, J. Brenner and Bernitsas (https://doi.org/10.1093/braincomms/fcae403) for a scientific commentary on this article.**

## Introduction

Primary mitochondrial diseases (PMDs) are a heterogeneous group of conditions caused by pathogenic genetic variants in mitochondrial or nuclear genes that impact oxidative phosphorylation, a process vital for energy production in cells.^1^ PMDs affect organs with high energy demands, such as the central nervous system, due to their reliance on efficient energy metabolism.^2^ The metabolic profile of PMD is marked by compromised ATP production and an increase in reactive oxygen species (ROS), leading to further damage to cellular components.^3^

One of the less explored but highly impactful symptoms of these diseases is auditory impairment. Hearing loss is frequently reported in PMD,^4-9^ reflecting the susceptibility of the entire auditory system to mitochondrial dysfunction. The complexity of hearing loss in people with PMD is shown by the frequent reports in specialist clinics that traditional hearing aids are ineffective. Mitochondrial function is critical for generating the endolymphatic potential within the cochlea^10^ that drives the transduction processes of hair cells^11^ and thus integral to the inner ear’s operation.^12,13^ Beyond the ‘sensory’ type hearing loss,^4,6^ hearing impairment in PMD may also have neural components.^7,14^ For example, the pathogenic m.3243A>G variant, often associated with Maternally Inherited Deafness and Diabetes (MIDD) and Mitochondrial Encephalopathy Lactate Acidosis and Stroke-like episodes (MELAS), has been shown to contribute to both cochlea dysfunction and auditory nerve impairment, as suggested by previous auditory phenotyping research.^4,15^ Auditory neuropathy is commonly associated with OPA1 missense variants,^14^ while mtDNA-related diseases frequently exhibit pronounced cochlea or mixed cochlea/neural dysfunctions.^4,6,7,14,16^ These studies have expanded our understanding of the variable impact mitochondrial dysfunction has on hearing. The hearing impairment ranges from mild to profound,^16,17^ often contributing to a progressive decline in communicative abilities that impact on daily social interactions, educational and professional activities and degrade overall quality of life.^4^ The management of hearing impairment is therefore a critical component of the multidisciplinary approach required for the management of PMD.

Recent research by Santarelli *et al*.^7^ and Kullar *et al*.^6^ has made apparent that the pathophysiology of hearing loss in PMD is complex, involving multiple pathways and genetic factors. These studies, despite their insightful findings, were limited by small sample sizes comprising predominantly people with MELAS. Consequently, there is a need for precise genotype–phenotype correlations in larger, diverse cohorts to validate previous findings and enable more tailored approaches to the clinical assessment and treatment of hearing loss in PMD.

This study aims to deepen current understanding of auditory dysfunction in PMD through a comprehensive analysis of auditory processing in a large, genotypically heterogenous group. Our diverse cohort has enabled investigation of the complex interplay between different pathogenic PMD-related genetic variants and their unique auditory phenotypes. The research is driven by two primary objectives: first, to determine the degree to which pathogenic PMD-related genetic variants affect cochlea and neural mechanisms; and second, to understand how these effects are reflected in the audiological profiles of individuals with PMD. We hypothesized that a spectrum of auditory dysfunctions exists, potentially exacerbating the functional hearing impairment. By exploring the association between genotype and phenotypic hearing impairment in patients with PMD, our study seeks to clarify the broader implications of mitochondrial genetics in auditory function with the goal of informing personalized clinical management strategies that are attuned to the complexities of mitochondrial genetics and auditory rehabilitation.

## Materials and methods

### Participants, study design and ethical considerations

We conducted a cross-sectional observational study on adults with a genetic or clinicopathological diagnosis of PMD attending the London NHS England Highly Specialised Service for Rare Mitochondrial Disorders clinic. The study involved individuals aged 16 and above, with and without self-reported hearing impairment, to explore the prevalence, subtype and degree of auditory impairments associated with *PMD*. Those with recent local ear infection were excluded. We included objective auditory assessments in our study to mitigate the likelihood of underreporting and to ensure a detailed evaluation of the true prevalence of hearing impairments in this population.^18^ Patients were categorized based on their genetic results: m.3243A>G/T mutation, other pathogenic mtDNA mutations, nuclear gene variants associated with PMD and clinicopathological diagnosis of PMD. This study received approval from the institutional review board (IRB approval no. 20/YH/0014). Prior to participation, informed consent was obtained.

### Data collection methodology

Due to the COVID-19 pandemic, our study necessitated methodological adjustments to ensure participant safety and compliance with public health guidelines. Auditory tests were conducted in our audiovestibular laboratory or at home, the latter for participants deemed at high risk for serious illness from COVID-19.^19^ We ensured the privacy and comfort of all participants, especially when conducting assessments at home. We included a verification process for home-based testing to confirm its reliability, with a subset of participants undergoing a comparative in-hospital test.^20^ This dual-setting approach allowed us to cross-verify the audiological outcomes, ensuring consistency and accuracy. Additionally, this methodology offered an opportunity to explore the viability of remote audiological evaluations, a subject that is further discussed in Male *et al*.^20^ The same examiner (N.K.) conducted all tests, and the same equipment was used for both home and hospital visits to ensure consistency in the audiological assessments.

### Audiological assessment

Participants underwent a detailed audiological evaluation with tests specifically chosen to provide information on the type and severity of hearing loss, and to differentiate between cochlea, neural and/or pathologies distal to the auditory nerve. Audiological assessments are summarized in Table 1.

**Table 1:** Overview of audiological assessments and neurophysiological tests conducted for evaluating auditory function

Test	Equipment	Procedure	Test parameters	Clinical significance
Pure-tone audiometry (PTA)	Calisto audiometer	Air and bone conduction threshold determination	Air: 0.25–8 kHz, Bone: 0.5–4 kHz; calibration to ISO standards	Assesses auditory threshold, calculates average thresholds for 4- (0.5, 1, 2, 4 kHz) and high frequencies (4 and 8 kHz)
Tympanometry	Titan™ system	Middle ear function assessment	Type A, As, Ad, B or C (Jerger classification)	Classifies middle ear conditions, such as effusion or Eustachian tube dysfunction
Acoustic reflex thresholds (ART)	Titan™ system	Bilateral reflex elicitation	Lowest level of reflex elicitation	Indicates possible pathologies, including middle and inner ear issues and nerve/brainstem disorders
Quick speech-in-noise test (QSiN)	Calisto audiometer	Speech-in-noise comprehension ability assessment	SNR loss determination	Evaluates ability to perceive speech in the presence of background noise
Listening in spatialized noise-sentences test (LiSN-S)	Phonak software, Sennheiser HD 215 headphones	Speech perception in directional noise assessment	DV90, SV90, HC and LC conditions; ‘spatial advantage’ and ‘talker advantage’ measures	Measures spatial processing abilities assessing the utilization of interaural differences and allows for the calculation of a spatial processing disorder (SPD) pattern. SPD is characterized by impaired ability to allocate attention to speech from different locations, especially in cocktail party conditions
Auditory brainstem responses (ABRs)	Interacoustics Eclipse system	Auditory nerve pathways and brainstem functionality examination	Clicks with the rates of 11.1; absolute and interpeak wave I–V latencies	Identifies auditory pathway dysfunction from the distal portion of auditory nerve to the midbrain
Distortion product otoacoustic emissions (DPOAEs)	Interacoustics Eclipse system	Outer hair cells function within the cochlea evaluation	f1 and f2 tone presentation; 2f1-f2 frequency analysis	Assesses cochlea (outer hair cell) health, differentiates cochlea from other auditory pathologies

### The Montreal Cognitive Assessment (MoCA)

One important everyday skill for auditory perception is speech perception, which is related to cognition^21^ and linked with working memory measurements.^22^ However, the accuracy of some cognitive measurements may be negatively influenced by auditory sensory system dysfunction.^23^ To control for confounding cognitive factors, we collected some information on the cognitive status of patients using the Montreal Cognitive Assessment (MoCA BLIND version 8.2),^24^ which is a brief screening test that assesses a wide range of cognitive functions sensitive to mild cognitive impairment. MoCA includes sections on attention and concentration, memory, language, conceptual thinking, calculations and orientation (22-point test cut-off of <18/22 for mild cognitive impairment).

### The Newcastle Mitochondrial Disease Adult Scale (NMDAS)

The Newcastle Mitochondrial Disease Adult Scale (NMDAS)^25^ is a comprehensive tool that assesses various symptoms and functional impairments across multiple organ systems to evaluate the severity of mitochondrial disorders in adult participants. Participants were scored in domains such as neurological, muscular, visual and auditory functions, providing an overall severity score for an individual. This approach allowed for a standardized assessment of mitochondrial disease impact and progression in the adult population.

### Interpretation of auditory test results

We have developed a comprehensive classification system for hearing impairments, as detailed in Table 2. This system was established to address a need for greater specificity in auditory diagnosis. The criteria for our classification are informed by a thorough review of the literature, as well as the clinical expertise of our interdisciplinary team. It is designed to include a wide range of auditory evaluations, including pure-tone audiometry (PTA) for air and bone conduction thresholds, the quick speech-in-noise (QSiN) test for speech-in-noise discrimination, tympanometry to assess middle ear function, distortion product otoacoustic emission (DPOAE) testing to evaluate cochlea function, the listening in spatialized noise-sentences test (LiSN-S) for assessing auditory spatial processing ability and auditory-evoked brainstem response (ABR) measurements for neurological evaluation from the distal portion of auditory nerve to the midbrain.

**Table 2:** Classification of auditory impairments: criteria and observations

Type of impairment	Diagnostic criteria	Additional comments
No hearing impairment	No abnormalities across auditory tests	Normal function on all assessments
Peripheral hearing impairment	Elevated thresholds in four-frequency or high-frequency average (>20 dB as per BSA recommended procedure, 2018); normal tympanometry and ABR; deficits in QuickSIN; abnormal otoacoustic emissions; absent or elevated ART*	Indicative of cochlea issues
Neural/central auditory involvement	Normal pure-tone thresholds (≤20 dB at 0.5, 1.0, 2.0 and 4.0 kHz); presence of otoacoustic emissions; normal tympanometry; abnormal ART and/or ABR; SPD pattern in LiSN-S test	Suggests neural and/or distal to the auditory nerve pathologies including auditory brainstem generators
Mixed auditory involvement	Signs of both peripheral deficits and neural/central processing deficits	Indicates combined cochlea and neural, and/or brainstem dysfunctions
Inconclusive hearing impairment	Inability to complete the full range of tests due to severe to profound hearing loss	Results not sufficiently conclusive for categorization

^*^Further analysis with ART considered for potential central/neural auditory patterns.

Our classification categorizes auditory impairments into—‘No Hearing Impairment’, ‘Peripheral Hearing Impairment’, ‘Neural/Central Auditory Involvement’, ‘Mixed Auditory Involvement’ and ‘Inconclusive Hearing Impairment’. Each category is defined by specific diagnostic criteria and observations, derived from objective clinical assessments, as outlined in Table 2.

All individuals diagnosed with neural/central or mixed auditory impairments were referred to a specialist in audiovestibular medicine (D-E.B.) who has extensive expertise in auditory processing disorders. The specialist, who was blind to the initial assessment results, then confirmed the type of hearing impairment, ensuring an accurate diagnosis and the formulation of an appropriate intervention strategy.

### Statistical analysis

Descriptive statistics were compiled for baseline characteristics and auditory test results, which included means, standard deviations and medians. To determine the influence of different genetic groups (m.3243A>G/T group, nuclear gene variants, other pathogenic mtDNA variants and a clinicopathological diagnosis) on auditory performance while adjusting for age, cognitive status, hearing level and disease severity, a multivariate analysis of covariance (MANCOVA) was conducted. The independent variable for the MANCOVA was the diagnostic group, and the dependent variables comprised ear specific PTA and QSiN scores, and all measures of LiSN-S tests.

Levene’s test for equality of variances was applied to check the assumption of homogeneity across the groups for each auditory measure. Following a significant MANCOVA outcome, pairwise comparisons with Bonferroni correction were performed to establish differences between the diagnostic groups.

Effect sizes for the MANCOVA were reported to quantify the magnitude of differences observed between groups, and power analyses were carried out *post hoc* to determine the study’s capacity to detect meaningful effects. Furthermore, multinomial logistic regression was employed to explore the relationship between pathogenic genetic variants and the likelihood of abnormal auditory test results, yielding odds ratios and 95% confidence intervals.

Nested models were evaluated, incrementally adding variables to each subsequent model. Model fit was compared using the Akaike information criterion (AIC) and Bayesian information criterion (BIC), and the optimal model was selected based on these criteria.

All statistical analyses were performed using Microsoft Excel and SPSS version 29. A *P*-value threshold of <0.05 was set for statistical significance.

## Results

### Participant demographics and diagnostic groups

Of the initial 83 adults recruited for the study, 74 consented to participate. After excluding two individuals with cochlear implants, our analysis included a final cohort of 72 patients. The age range of participants was 20–81 years old, with an average age of 48.26 years (SD ± 15.15 years), consisting of 49 females and 23 males. Molecular diagnoses varied, with the most prevalent being the m.3243A>G pathogenic variant (*n* = 40) and one m.3243A>T pathogenic variant, collectively representing 56% of the group (as participants with m.3243A>G and m.3243A>T were phenotypically similar, we analyzed both together and refer to all as our m.3243A>G/T group). Other pathogenic mitochondrial DNA variants (including other single nucleotide variants and single large-scale deletions of the mtDNA) were present in 18 participants (26%), nuclear gene variants (two *POLG*, one *OPA1*, one *PEO1*, one *RNASEH1*, one *SURF1*) in six (8%), and seven participants (10%) had a clinicopathological diagnosis without genetic confirmation. The median NMDAS score was 12.5, with an interquartile range of 11.75 (from 6.25 to 18).

Regarding the distribution of testing due to COVID-19 adaptations, 55 participants were tested in our audiovestibular laboratory. The remaining 17 participants, identified as high risk for COVID-19 complications, underwent testing at home. To ensure the reliability of the home-based audiological assessments, we performed a cross-verification with in-hospital tests for a subset of seven participants from the at-home group.^20^ Detailed diagnostic information is provided in Tables 3 and 4 and Supplementary Table 1.

**Table 3:** Presents the number of individuals (count), the average age (mean age) and the age standard deviation (age SD) within each diagnostic group

Diagnostic group	Sample size	Mean age	Age SD
m.3243A>G/T (m.3243A>G, *n* = 40 and m.3243A>T, *n* = 1)	41	48.60	14.28
Nuclear gene variants (*POLG* (2), *OPA1* (1), *PEO1* (1), *RNASEH1* (1), *SURF1* (1))	6	52.71	15.55
Clinicopathological diagnosis	7	58.17	19.56
Other pathogenic mtDNA variants	18	43.89	13.39

**Table 4:** Descriptive statistics of auditory test results by diagnostic group for PMD patients

Diagnostic group	ART %, (*n*)	ABR %, (*n*) left	ABR %, (*n*) right	LiSN-S pattern % and (*n*)	DPOAE left % and (*n*)	DPOAE right % and (*n*)
m.3243A>G/T (41)	Normal: 30% (11) Abnormal: 70% (26) Missing: 4	Normal: 32% (12) Abnormal: 68% (26) Missing: 3	Normal: 34% (13) Abnormal: 66% (25) Missing: 3	SPD: 82% (27) Other/normal: 18% (6) Missing: 8	Present: 34%(13) Absent: 66%(25) Missing: 3	Present: 32% (12) Absent: 68% (26) Missing: 3
Nuclear gene variants (6)	Normal: 60% (3) Abnormal: 40% (2) Missing: 1	Normal: 29% (2) Abnormal: 71% (4) Missing: 0	Normal: 33% (2) Abnormal: 67% (4) Missing: 0	SPD: 33% (2) Other/normal: 67% (4) Missing: 1	Present: 60% (3) Absent: 40% (2) Missing: 1	Present: 60% (3) Absent: 40% (2) Missing: 1
Clinicopathological diagnoses (7)	Normal: 57% (4) Abnormal: 43% (3)	Normal: 50% (3) Abnormal: 50% (3) Missing: 1	Normal: 50% (3) Abnormal: 50% (3) Missing: 1	SPD: 67% (4) Other/normal: 33% (2) Missing: 1	Present: 71% (5) Absent: 29% (2)	Present: 71% (5) Absent: 29% (2)
Other pathogenic mtDNA variants (18)	Normal: 56% (9) Abnormal: 44% (7) Missing: 2	Normal: 73% (11) Abnormal: 27% (4) Missing: 3	Normal: 80% (12) Abnormal: 20% (3) Missing: 3	SPD: 56% (9) Other/normal: 44% (7) Missing: 2	Present: 65%(11) Absent: 35% (6) Missing: 1	Present: 59% (10) Absent: 41% (7) Missing: 1
Overall (72)	Normal: 42% (27) Abnormal: 58% (37) Missing: 8	Normal: 48% (31) Abnormal: 52%(34) Missing: 7	Normal: 51% (33) Abnormal: 49% (32) Missing: 7	SPD: 69%(42) Other/normal: 31% (19) Missing: 11	Present: 48%(32) Absent: 52%(34) Missing: 6	Present: 45%(30) Absent: 56%(36) Missing: 6

This table presents the distribution of auditory test results across diagnostic groups in a cohort of PMD patients. The tests include auditory reflex threshold (ART), auditory brainstem response (ABR) for left and right ears, the LiSN-S pattern and distortion product otoacoustic emissions (DPOAE) for both ears. Results are categorized into normal and abnormal findings, with additional details on presence/absence and missing data noted.

### Comparative analysis of auditory function across diagnostic groups in PMD

We investigated the differences in auditory function across four diagnostic groups of mitochondrial disease: m.3243A>G/T group, nuclear gene variants, other pathogenic mtDNA variants and a clinicopathological diagnosis (for more detailed information on the grouping and genetic variants, please refer to Supplementary Table 1). A MANCOVA was conducted with several auditory tests, controlling for covariates like age, cognitive status (via the MoCA) and disease progression (through the NMDAS).

#### MANCOVA results and pairwise comparisons

**Figure 1:**
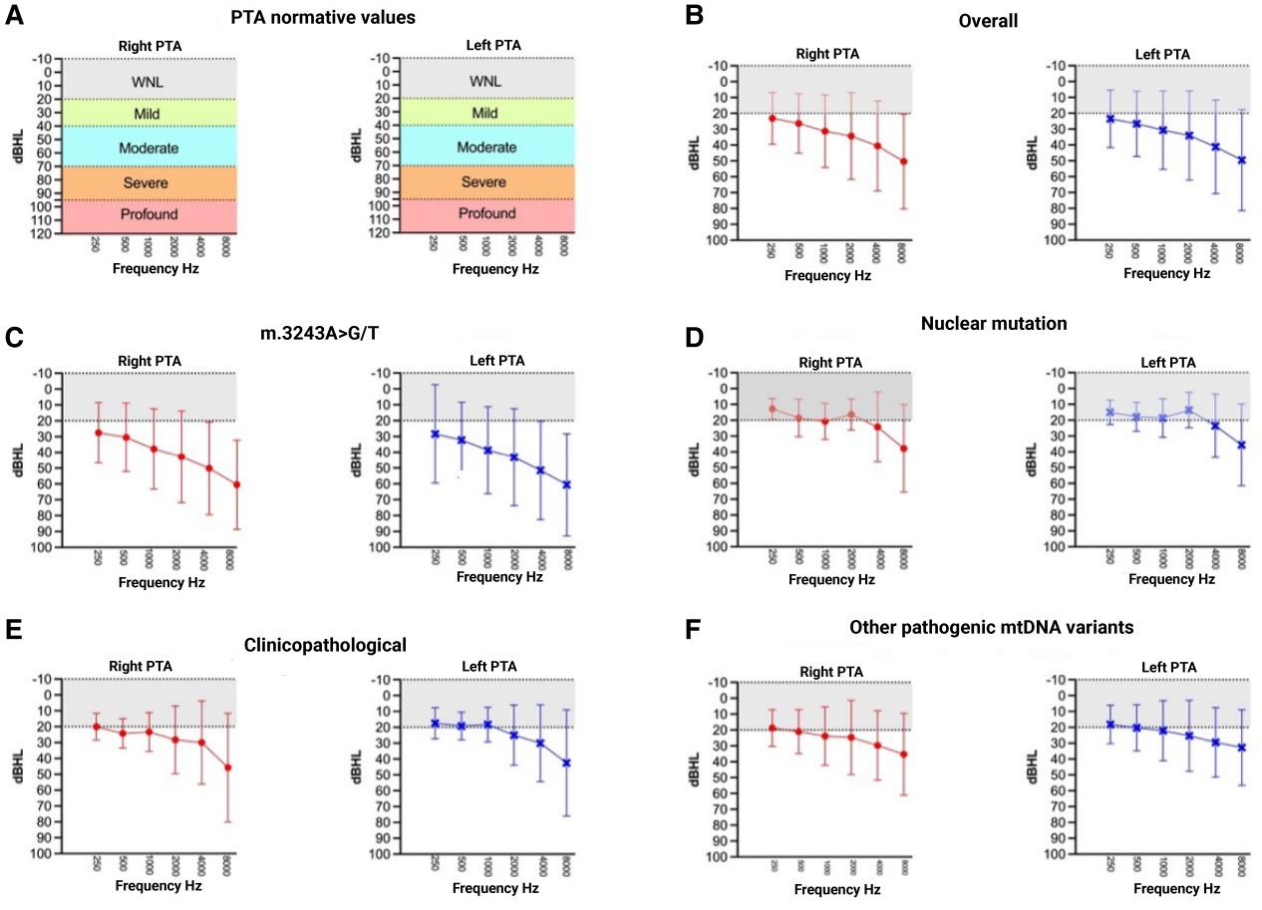
**Pure-tone audiometry (PTA) results in primary mitochondrial disease (PMD) patients.** Panel **A** displays normative values for right and left PTA, categorizing hearing threshold levels from within normal limits (WNL) to profound hearing loss (BSA, 2018). Panels **B–F** represent PTA thresholds at different frequencies for the right and left ears of PMD patients. Panel **B** shows overall PMD patient data, while panels **C–F** illustrate results stratified by genetic mutations: m.3243A>G/T (3243), nuclear gene variant, clinicopathological and other pathogenic mtDNA variants, respectively. Error bars represent standard deviations, indicating the range of hearing thresholds within each subgroup. The shaded areas behind the data points reflect the degree of hearing loss as defined in panel **A**. The 3243 group in panel **C** (*) demonstrated the highest average PTA scores across all measures, significantly differing from the other groups. A multivariate analysis of covariance (MANCOVA) was used to compare the PTA thresholds between different genetic mutation groups while controlling for age, cognitive status and disease severity. Statistics values: *F*(3,59) = 7.115, *P* < 0.001 for average air-conduction PTA left; *F*(3,59) = 7.882, *P* < 0.001 for average air-conduction PTA right. Sample sizes: m.3243A>G/T group (*N* = 36), nuclear mutation (*N* = 7), nuclear mutation (excluding mt gene) (*N* = 5), other pathogenic mtDNA variants (*N* = 18). Although the nuclear mutation groups have fewer than 10 samples, individual data points are not shown to maintain consistency across all panels. Graphs with individual data points for these groups are provided in the Supplementary material (Supplementary Fig. 1). Created in BioRender. Koohi, N. (2024) https://BioRender.com/f10a584.

We found significant differences (at *P* < 0.05) in the average PTA thresholds and high-frequency averages across all diagnostic groups compared to the m.3243A>G/T group, suggesting raised auditory thresholds associated with this mutation. These differences were statistically significant with substantial effect sizes and observed power, indicating a robust difference between groups (see detailed analysis below). The comprehensive results of PTA across the study cohort are shown in Fig. 1.

**Figure 2:**
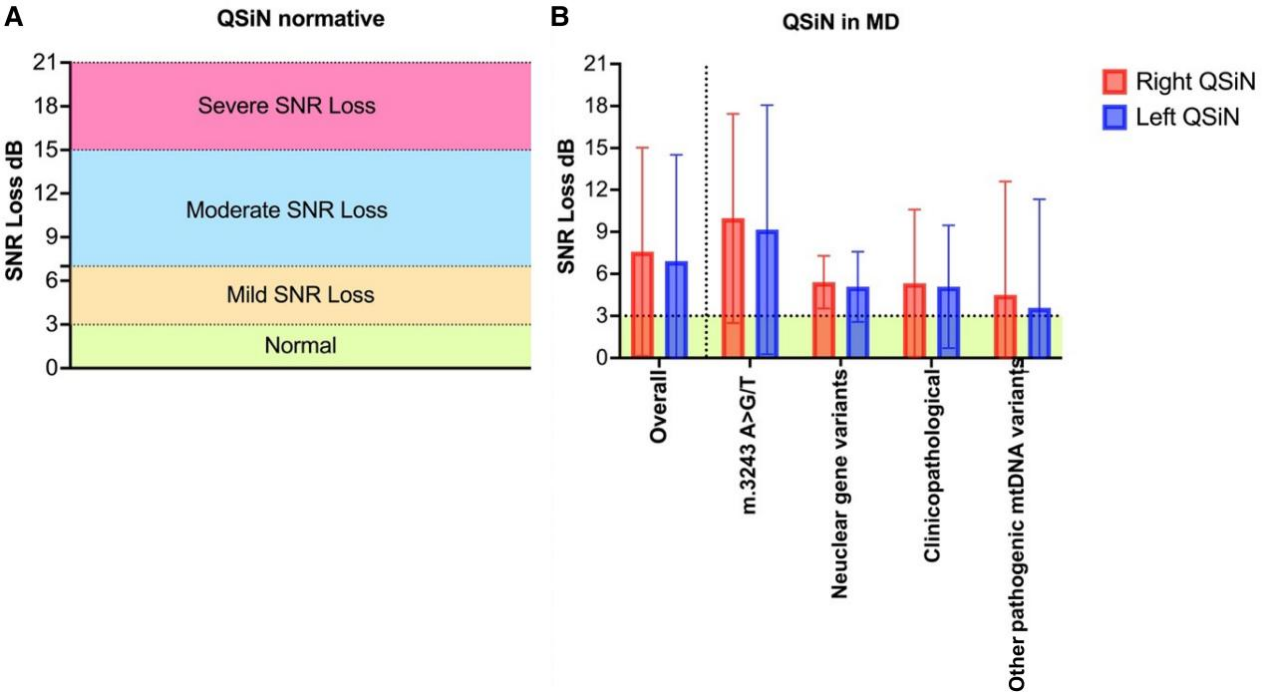
**Quick speech-in-noise (QSiN) performance in primary mitochondrial disease (PMD) patients compared to normative data.** Panel **A** represents the normative data for the QSiN test, categorizing the signal-to-noise ratio (SNR) loss into normal, mild SNR loss, moderate SNR loss and severe SNR loss. Panel **B** compares the SNR loss in mitochondrial disease patients, with separate bars for right and left ear performance across different genetic mutations: overall, m.3243A>G/T, nuclear gene variants, clinicopathological diagnoses and other pathogenic mitochondrial mtDNA variants. The error bars indicate the variability within each subgroup. The horizontal dotted lines across panel **B** indicate the normative data SNR loss range. The vertical dotted line in panel **B** separates the overall QSIN results for the entire PMD cohort from the specific diagnostic subgroups. Statistical test used: MANCOVA (QSiN SNR left: *F*(3,50) = 0.783, *P* = 0.509; QSiN SNR right: *F*(3,50) = 0.450, *P* = 0.719). Sample sizes: m.3243A>G/T group (*N* = 28), nuclear gene variants (*N* = 6), nuclear gene variants (excluding mt gene) (*N* = 5), other pathogenic mtDNA variants (*N* = 15). Although the nuclear mutation groups have fewer than 10 samples, individual data points are not shown to maintain consistency across all panels. Graphs with individual data points for these groups are provided in the Supplementary material (Supplementary Fig. 1). Created in BioRender. Koohi, N. (2024) https://BioRender.com/b98j678.

Figure 2 shows QSiN test results per PMD subgroup compared to normative data. There were no significant differences in QSiN scores for either ear across diagnostic groups when controlling for covariates.

**Figure 3:**
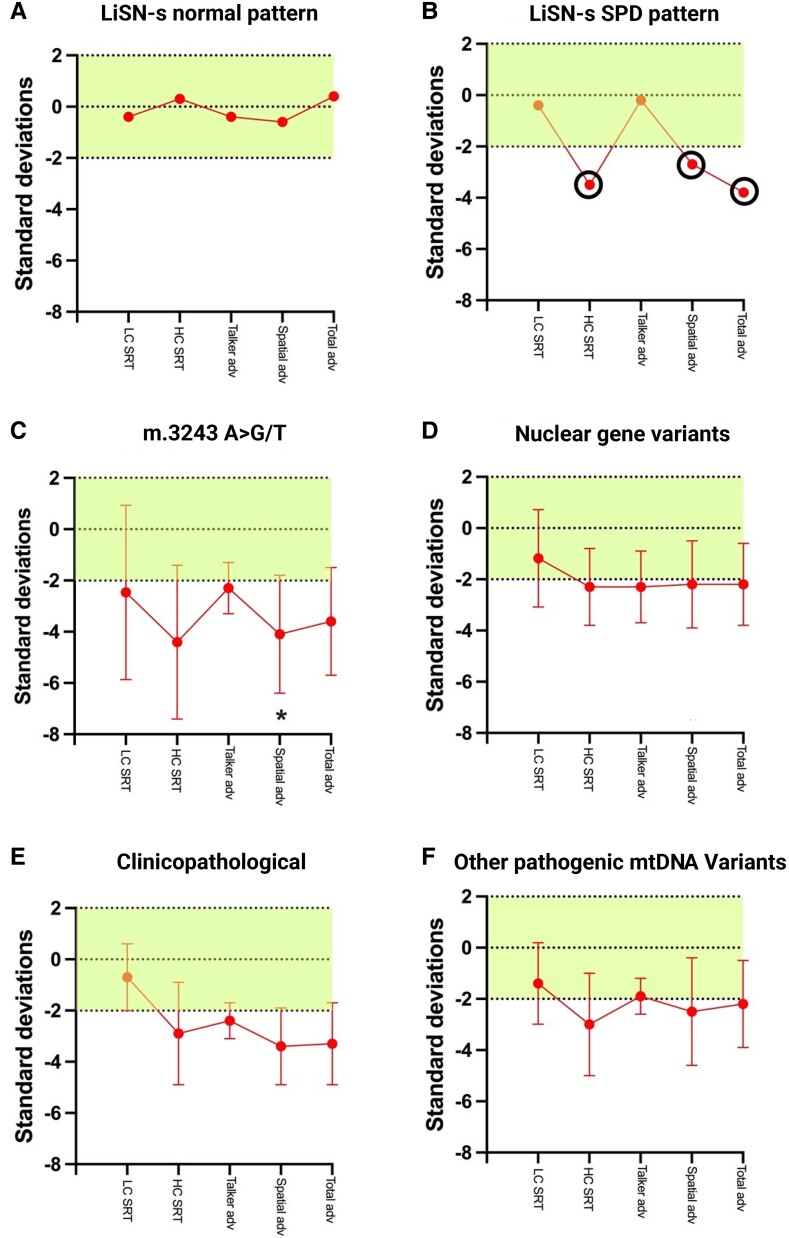
**Spatial auditory processing performance in mitochondrial disease patients.** Panels **C–F** show the performance on the listening in spatialized noise-sentences test (LiSN-S) for mitochondrial disease patients with various genetic backgrounds. Standard deviations from the normative mean are plotted for different test conditions, including low cue (LC), high cue (HC), talker advantage (TA), spatial advantage (SA) and total advantage (TA). Positive values indicate performance above the normative population, while negative values indicate performance below. Panel **A** shows results for individuals with no spatial hearing impairment (normal LiSN-S pattern), while panel **B** presents results for patients with spatial processing disorder (SPD pattern). Panels **C** to **F** display results for patients from the following diagnostic groups: m.3243A>G/T, nuclear gene variants, other pathogenic mtDNA variants and clinicopathological diagnoses, respectively. Error bars represent standard errors, illustrating the variability within each patient group. A significant difference was only observed in the spatial advantage *Z*-score between the 3243 group (panel **C**) and the nuclear gene variants group (MANCOVA *F*(3,46) = 3.432, *P* = 0.025) (panel **D**), both marked with *, highlighting a specific deficit in spatial auditory processing for the 3243 group. Sample sizes: m.3243A>G/T group (*N* = 41), nuclear gene variants (*N* = 6), clinicopathological diagnosis (*N* = 7), other pathogenic mtDNA variants (*N* = 18), total sample for SPD (*N* = 61). Although the nuclear mutation groups have fewer than 10 samples, individual data points are not shown to maintain consistency across all panels. Graphs with individual data points for these groups are provided in the Supplementary material (Supplementary Fig. 1). Created in BioRender. Koohi, N. (2024) https://BioRender.com/a67e100.

Figure 3 details the cohort’s spatial auditory processing performance, with specific emphasis on the challenges faced by different diagnostic groups. For the LiSN-S, our analysis revealed a significant difference in the spatial advantage *Z*-score (*F*(3,46) = 3.432, *P* = 0.025, partial η² = 0.183), between the m.3243A>G/T group and the nuclear gene variants group. No significant differences were observed for the low cue *Z*-score (*P* = 0.859), high cue *Z*-score (*P* = 0.628), talker advantage *Z*-score (*P* = 0.567) or total advantage *Z*-score (*P* = 0.491).

#### Effect sizes and power

The effect sizes for each significant comparison ranged from moderate to large, emphasizing the practical importance of these genetic differences in auditory function. The statistical power for these tests was high (>0.99), indicating that our sample size was sufficient to detect these differences.

#### Categorical data

The logistic regression analysis showed a strong fit with the observed data (−2 Log likelihood: 33.914, chi-square: 44.759, df = 6, *P* < 0.001), indicating significant predictive capacity. This supported by Cox and Snell and Nagelkerke R Square values of 0.538 and 0.724, respectively. Diagnostic group 1 (m.3243A>G/T) had a significantly higher likelihood of abnormal acoustic reflex thresholds (B = 4.108, *P* = 0.003; Exp(B) = 0.016, 95% CI [0.001, 0.255]).

In our logistic regression analysis examining the association between diagnostic groups and the likelihood of ABR abnormalities, we analysed a sample of 65 participants (with data missing for five participants). The model showed a strong fit with the data (−2 Log likelihood: 60.046, chi-square: 21.729, df = 6, *P* = 0.001), and moderate to high explanatory power (Cox and Snell R Square = 0.308, Nagelkerke R Square = 0.411). Our analysis revealed that individuals in diagnostic group 1 (m.3243A>G/T) had a significantly higher likelihood of ABR abnormalities (B = 2.595, *P* = 0.003; 95% CI [2.350, 76.326]), with an odds ratio of 13.393 compared to the reference category. This effect was notably different from other diagnostic groups, which did not show significant associations with ABR abnormalities. Among the control variables, the sum of completed NMDAS questions (25 items) displayed a moderate influence (B = 0.098, *P* = 0.040; 95% CI [1.004, 1.210]), while age, MoCA raw scores and hearing thresholds at frequencies 2000 and 4000 Hz did not show significant effects.

We used logistic regression to examine the influence of diagnostic groups on the likelihood of having SPD pattern in the LiSN-S test, controlling for variables including age, NMDAS scores, MoCA raw scores and average PTA in the worse ear. This analysis included data from 55 individuals who completed the LiSN-S test (covering 76% of our study cohort). The model demonstrated moderate to strong explanatory power (−2 Log likelihood: 35.845, Cox and Snell R Square = 0.443). Diagnostic grouping was a significant factor in predicting SPD occurrence. Specifically, individuals in the m.3243A>G/T group had a significantly higher likelihood of SPD (B = 2.389, *P* = 0.035), as shown by the Wald chi-square test. Other diagnostic groups did not show significant differences. Notably, age, NMDAS scores, average PTA in the worse ear and MoCA scores were not significant predictors in this model.

### Relationship between genetic diagnosis and hearing categories: a multinomial logistic regression analysis

**Figure 4:**
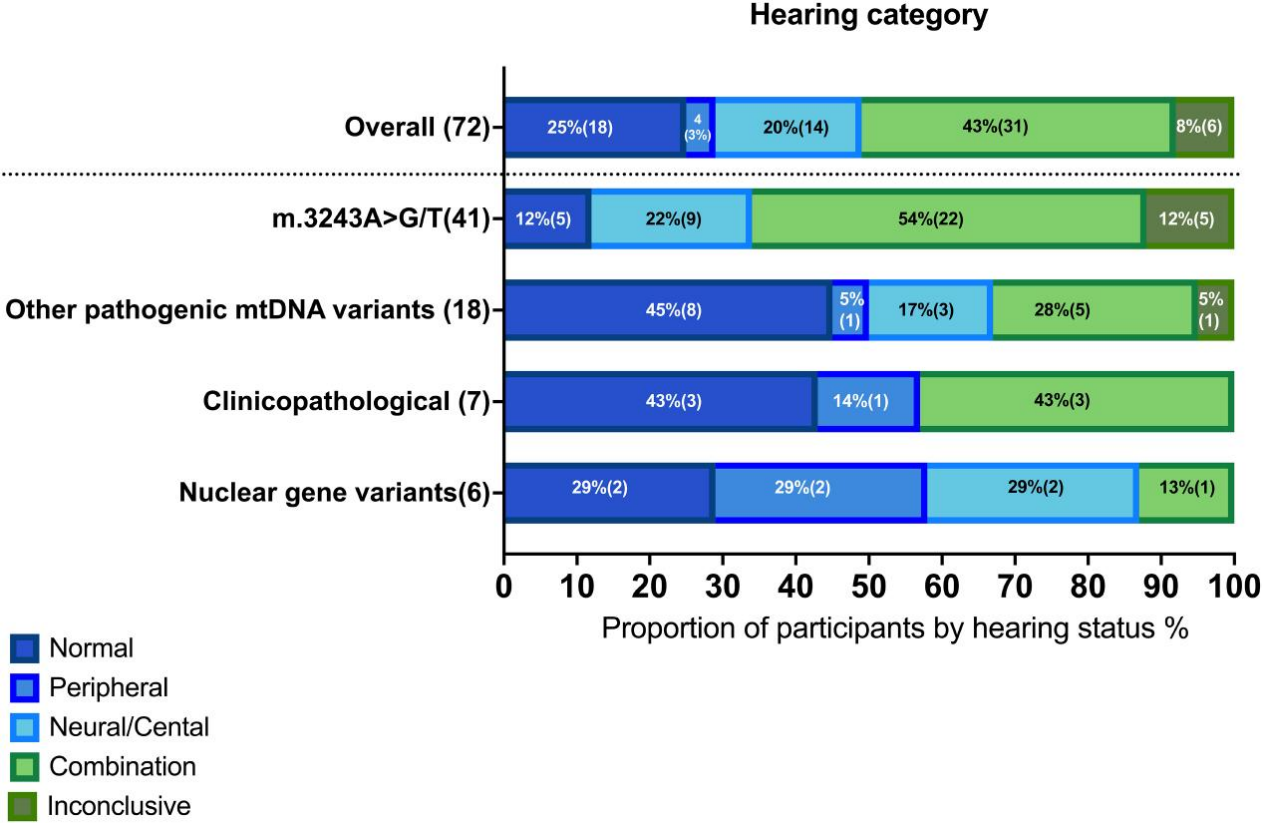
**Prevalence of hearing impairment types across diagnostic groups.** Distribution of hearing impairment types including no impairment, neural/central, peripheral, peripheral and neural/central and inconclusive across different diagnostic groups identified in mitochondrial disease patients. Each bar represents the percentage of individuals within a diagnostic group exhibiting a specific type of hearing function or impairment. Created in BioRender. Koohi, N. (2024) https://BioRender.com/v37y429.

The distribution of hearing impairment types across diagnostic groups is shown in Fig. 4. We used multinomial logistic regression to assess the influence of genetic mutations, age and disease severity on hearing impairment. We constructed and compared several models, ultimately identifying one that incorporated genetic factors and NMDAS scores as most predictive.

For comprehensive model statistics and their comparative analyses, please see the Supplementary Information. This includes the associations of NMDAS scores with hearing impairment across diagnostic groups, where diagnostic group 1 (m.3243A>G/T) showed notable correlations.

In Table 5, we present the odds ratios and their 95% confidence intervals for each diagnostic group across different hearing categories. These results indicate significant associations in certain categories, particularly for diagnostic group 1 (m.3243A>G/T).

**Table 5:** This table delineates the strength of association between diagnostic groups and various hearing categories through odds ratios (Exp(B)) and their corresponding 95% confidence intervals (CI)

Hearing category	Diagnostic group	Odds ratio Exp(B)	95% CI	*P*-value
Category 2 (neural/central)	Group 1 (m.3243A>G/T)	1.898	1.100–3.277	0.021*
Category 2 (neural/central)	Group 2 (nuclear gene variants)	1.070	0.936–1.224	0.319
Category 2 (neural/central)	Group 3 (clinicopathological)	1.043	0.882–1.233	0.626
Category 3 (peripheral)	Group 1 (m.3243A>G/T)			
Category 3 (peripheral)	Group 2 (nuclear gene variants)	1.013	0.845–1.215	0.890
Category 3 (peripheral)	Group 3 (clinicopathological)	0.915	0.680–1.231	0.557
Category 4 (peripheral and neural/central)	Group 1 (m.3243A>G/T)	2.255	1.308–3.887	0.003*
Category 4 (peripheral and neural/central)	Group 2 (nuclear gene variants)	1.171	1.039–1.320	0.010*
Category 4 (peripheral and neural/central)	Group 3 (clinicopathological)	1.192	1.039–1.368	0.012*
Category 5 (inconclusive)	Group 1 (m.3243A>G/T)	2.258	1.301–3.918	0.004*
Category 5 (inconclusive)	Group 2 (nuclear gene variants)			
Category 5 (inconclusive)	Group 3 (clinicopathological)	1.144	0.944	0.657

The *P*-values indicate the level of statistical significance for each association. Statistically significant values are marked with an asterisk (*). Notable findings include significant associations for diagnostic group 1 in categories 2, 4, and 5, with odds ratios suggesting an increased risk or prevalence in these hearing categories. Diagnostic group 4 (other pathogenic mtDNA variants) was used as the reference category.

## Discussion

Our study provides novel insights into the auditory disturbance associated with PMD. We show that mitochondrial dysfunction manifests in diverse auditory deficits, ranging from subtle cochlea impairments to pronounced auditory processing deficits, varying across different genotypes. These observations underline the importance of detailed auditory assessments in patients with PMD to tailor clinical approaches effectively, and explains why many people with PMD seen in clinic report no benefit from traditional hearing aids.

We observed that patients with the pathogenic m.3243A>G/T variant exhibit a wide array of auditory deficits that included the cochlea, but also extended to the neural/central auditory processing areas, as was suggested in previous studies.^7^ The significant hearing threshold elevations and spatial processing deficits identified in our m.3243A>G/T group highlight a profound impact on hearing abilities in affected individuals and the advantages of a genotype-specific approach to auditory assessment and management. Compared to previous studies,^6,8,16,26,27^ which have traditionally centred on cochlea dysfunction, our research extends current understanding and reveals a more complex, multifactorial basis to PMD-related hearing loss, with neural/central auditory dysfunction contributing towards 63% of hearing loss in our cohort.

Analysis of audiological evaluations across different pathogenic genetic variants confirmed that the m.3243A>G/T group had significantly higher PTA thresholds, especially at high frequencies, compared with other genetic groups.^28^ The high metabolic demand of the cochlea necessitates robust mitochondrial function for energy production and calcium homeostasis, critical for hair cell function. Dysfunctional mitochondria, as seen with the m.3243A>G/T group, lead to disruptions in normal metabolic processes, such as reduced ATP production, elevated ROS levels and impaired calcium homeostasis. These factors contribute to oxidative stress, which subsequently causes cochlear damage and hearing loss.^13,29^ The statistical significance of these findings, alongside substantial effect sizes and observed power, confirm clear auditory differences associated with this pathogenic variant. In addition, we observed that the shape of the audiogram varied among the different genetic groups. This variability in audiogram shapes across groups suggests the diverse impact of mitochondrial dysfunction on different cochlear structures.^30^

The increased PTA thresholds in the m.3243A>G/T group indicate a specific vulnerability in auditory perception. Importantly, while QuickSiN test score abnormalities were universally present across PMD, suggesting a widespread difficulty in speech-in-noise perception, the LiSN-S test revealed a distinct spatial processing deficit (i.e. notable deviation in the spatial advantage *Z*-score exclusively in the m.3243A>G/T group after accounting for confounding factors such as age, NMDAS levels, and hearing loss). Conversely, other measures assessed by the LiSN-S test did not exhibit significant variations across the different genotypes, further emphasizing the unique impact of the m.3243A>G/T variant on spatial processing within auditory function.

In our audiological assessments, we focused on the SPD (spatial processing disorder) pattern, a constellation of auditory processing challenges that signal a broader, more widespread disorder (for more information, refer to Cameron *et al*.^31^). This assessment includes several components, including the spatial advantage measure, which is a specific ability to distinguish target sounds in the presence of background speech in a spatial context (i.e. ‘cocktail party’ condition). We found that the m.3243A>G/T variant influenced the spatial advantage measure, and also contributed more broadly towards spatial auditory processing. While the spatial advantage measure, notably different in the m.3243A>G/T group, is a single aspect of the SPD pattern, our logistic regression analysis revealed that this genetic variant considerably heightens the risk of SPD. The SPD pattern, which includes the spatial advantage measure, was significantly more prevalent in individuals with the m.3243A>G/T variant. It is critical to distinguish between the two; while the spatial advantage measure is a singular metric, the SPD pattern represents a range of auditory processing challenges that indicate a more widespread disorder.

Our investigation into SPD abnormalities, especially in patients with m.3243A>G/T variant, aligns with previous work that has shown impaired interaural latency differences (ILD) in MELAS, where increased ILD suggest altered auditory conduction times from potential brainstem generator dysfunction.^7^ Complementarily, our cohort exhibited a similar prevalence of ABR abnormalities, confirming a widespread pattern of auditory processing disruption. This disruption concurs with mitochondrial dysfunction and correlates with pronounced spatial processing deficits observed in our SPD data. Furthermore, existing research has definitively linked neural auditory pathway pathologies with temporal processing and binaural integration challenges,^32^ in addition to brainstem pathologies with auditory spatial processing difficulties.^33-35^ These collective findings, alongside our ABR data, imply that the auditory processing challenges in PMD result from both peripheral and neural/central dysfunction.

Of particular interest, and serving to illustrate the natural history of auditory impairment in PMD, is the longitudinal case study of a patient in our cohort with the m.3243A>G variant. This patient’s auditory condition evolved from mild sensorineural hearing loss with poorly reproducible ABR to absent wave I, indicative of auditory nerve disruption, and further progressed to delayed interpeak latencies between waves III and V, with a subsequent delay in wave V and absent waves I and III over time. These observations document the dual impact of mitochondrial pathology on both the auditory nerve and brainstem generators. Moreover, within our cohort, instances of mild spatial processing deficits with normal ABR latencies, but poor reproducibility, highlight the diverse and complex presentation of auditory processing issues in PMD. This case, along with our broader findings, suggests that spatial auditory processing disorders in PMD may be linked to the multifaceted effects of mitochondrial dysfunction, underlining the need for a deeper understanding of the complex interplay between mitochondrial health and auditory processing in PMD.

To further delineate the impact of specific pathogenic mtDNA variants on auditory profiles and site of pathology, we used data from our detailed audiological assessments to systematically categorize hearing impairment into peripheral, neural, and/or central (neural/central) auditory processing involvement, or a combination thereof. Our findings suggest that genotyping may predict audiological phenotyping (Fig. 4). Incorporating disease severity, as measured using the NMDAS, further refined the predictive model, signifying that genetic profiles and disease severity offer a more accurate prediction of hearing impairment progression than age. The m.3243A>G/T variant was associated with greater severity in hearing impairment across both neural/central auditory pathways, and combined peripheral and neural/central categories, and appears to predispose individuals to severe auditory dysfunction that extends beyond the cochlea to involve neural and auditory processing centres. Conversely, nuclear gene variants displayed a negligible relationship with hearing impairment categories, with a mild association observed within combined peripheral and neural/central auditory impairments. This underlines the hypothesis that mitochondrial dysfunction, particularly those in our m.3243A>G/T group, can have extensive and profound consequences, disrupting auditory processing at both the sensory and neural levels.

In contrast, while the ‘other pathogenic mtDNA variant’ category did not show a statistically significant prevalence of neural/central or mixed hearing impairments, less than half of this group (42%) experienced such impairments. Although this finding did not reach statistical significance, it suggests that not all pathogenic mtDNA variants confer the same risk for neural/central auditory dysfunction, with the m.3243A>G/T conferring a particularly high risk.

Furthermore, some preliminary research has suggested the potential use of microRNA, such as miR-34a, as a biomarker for detecting inner ear damage in patients with mitochondrial disease.^36^ While further validation is needed, this approach could offer additional insights into monitoring sensorineural hearing loss in PMD.

### Limitations and future work

We recognize that our cohort represents one of the larger groups studied for the m.3243A>G/T variant in PMD, demonstrating the feasibility of gathering substantial participant numbers even for rare conditions. However, we also acknowledge that the diversity of pathogenic mtDNA variants is not fully captured in our current sample. The smaller group sizes for other mutations within our study highlight the ongoing need for larger and more diverse cohorts in future research, particularly focusing on these less represented PMD groups, to broaden the applicability of our findings and enhance our understanding of the full spectrum of PMD.

Although gender was initially included in the statistical analysis, it did not show any significant interactions or effects on the auditory outcomes. Consequently, gender was excluded from the final analysis to minimize the risk of multiple comparisons.

We recognize that the lack of MRI findings is a limitation of our study. While our primary objective was to investigate auditory impairments due to mitochondrial dysfunction, incorporating MRI in future research could offer additional insights into the structural and functional aspects of the central auditory nervous system, further enhancing our understanding of the relationship between mitochondrial disease and auditory dysfunction.

Subsequent research should focus on refining the precision of audiological testing methods. The current battery of tests provides valuable insights into the general location of auditory lesions, such as the cochlea or brainstem, but fell short of pinpointing the precise sites affected. Future investigations, equipped with advanced diagnostic tools such as electrocochleography, electrically-evoked compound action potential recordings and neurophonics or frequency-following responses, promise to localize lesions more accurately within these structures. Such precision will clarify the specific pathologies underpinning hearing impairments in PMD, enhancing our understanding of their aetiology and informing targeted treatment strategies.

Additionally, our cross-sectional approach provides a snapshot rather than the trajectory of auditory impairment. Longitudinal studies would be invaluable in understanding the rate and variability of hearing impairment development. The efficacy of auditory interventions, including hearing aids and cochlear implants, also merits further investigation to optimize clinical management strategies. Ultimately, the goal of future research should be to deepen our comprehension of the auditory implications of PMD and enhance life quality and clinical outcomes for those affected.

### Clinical implications

The discrepancy between the capacity to hear and the capacity to perceive speech emphasizes that the distortion of suprathreshold cues may be a pivotal factor in perceptual performance limitations.^32^ Our research highlights that mitochondrial dysfunction can lead to complex auditory phenotypes, which might go unnoticed in routine clinical evaluations. PTA, while foundational to hearing assessments, is not sensitive enough to detect auditory impairments beyond hearing sensitivity.

Interestingly, our findings revealed instances of mild SPD but normal ABR, with poor reproducibility. This illustrates the critical need to capture ‘hidden’ auditory impairments in early stages of the disease. Such early impairments can significantly influence a patient’s ability to process complex auditory information in daily environments.

Our research indicates that detailed auditory evaluations that identify neural/central auditory processing disorders, which frequently coexist with cochlea impairments in PMD, should be included as a part of a refined diagnostic approach in PMD. The neural/central auditory deficits identified in our study could explain the severe hearing challenges that people with PMD encounter, which are often inadequately captured by standard peripheral hearing assessments.^37,38^ Given these insights, it is imperative that clinical assessments are specifically tailored towards this patient group. Conventional hearing aids may not effectively address the neural/central components of their auditory processing difficulties, necessitating custom solutions.^39^ Furthermore, the long-term ineffectiveness of cochlear implants, as demonstrated by recent studies,^40^ suggests that implantation option may not be suitable for patients with progressive neural/central dysfunction associated with mitochondrial mutations. Thus, there is an urgent need for the development of innovative auditory rehabilitation strategies that address both cochlear and neural/central hearing impairments.

Beyond the scope of clinical practice, the implications of this study improve our understanding of the interplay between different PMD genotypes and auditory function. This knowledge serves a dual purpose; it is vital for the clinical management of PMD and for the development of specialized therapies and interventions aimed at enhancing the quality of life for those affected. Collaborative work involving geneticists, audiologists and neurologists is paramount to establishing multidimensional assessment protocols. These protocols should be capable of recognizing the complexities of auditory dysfunctions in PMD, considering both cochlea and neural contributions to hearing loss.

## Conclusion

In conclusion, our study reiterates the multifaceted nature of hearing impairment in PMD, emphasizing the involvement of both cochlea and neural/central auditory pathways. The strong association between specific pathogenic genetic variants and auditory profiles advocates for a personalized medicine approach to diagnosing and managing this patient population. Our findings lay the groundwork for future research to further elucidate the intricate relationships between mitochondrial dysfunction and the auditory system, aimed at improving the quality of life for individuals with PMD.

## Supplementary Material

fcae374_Supplementary_Data

## Data Availability

The data that support the findings of this study are available from the corresponding author upon reasonable request.

## References

[1] Pitceathly RD, Keshavan N, Rahman J, Rahman S. Moving towards clinical trials for PMD. J Inherit Metab Dis. 2021;44(1):22–41. 32618366 10.1002/jimd.12281 PMC8432143

[2] Poole OV, Hanna MG, Pitceathly RD. Mitochondrial disorders: Disease mechanisms and therapeutic approaches. Discov Med. 2015;20(111):325–331. 26645904

[3] Falabella M, Vernon HJ, Hanna MG, Claypool SM, Pitceathly RD. Cardiolipin, mitochondria, and neurological disease. Trends Endocrinol Metab. 2021;32(4):224–237. 33640250 10.1016/j.tem.2021.01.006 PMC8277580

[4] Sue CM, Lipsett LJ, Crimmins DS, et al Cochlear origin of hearing loss in MELAS syndrome. Ann Neurol. 1998;43(3):350–359. 9506552 10.1002/ana.410430313

[5] Kokotas H, Petersen MB, Willems PJ. Mitochondrial deafness. Clin Genet. 2007;71(5):379–391. 17489842 10.1111/j.1399-0004.2007.00800.x

[6] Kullar PJ, Quail J, Lindsey P, et al Both mitochondrial DNA and mitonuclear gene mutations cause hearing loss through cochlear dysfunction. Brain. 2016;139(Pt 6):e33. 27016405 10.1093/brain/aww051 PMC4892749

[7] Santarelli R, Cama E, Scimemi P, et al Reply: Both mitochondrial DNA and mitonuclear gene mutations cause hearing loss through cochlear dysfunction. Brain. 2016;139(6):e34. 27016406 10.1093/brain/aww052

[8] van Kempen CM, Beynon AJ, Smits JJ, Janssen MC. A retrospective cohort study exploring the association between different PMD and hearing loss. Mol Genet Metab. 2022;135(4):333–341. 35190254 10.1016/j.ymgme.2022.02.003

[9] Cadoni G, Primiano G, Picciotti PM, et al Hearing impairment and neuroimaging results in PMD. J Pers Med. 2023;13(9):1329. 37763097 10.3390/jpm13091329 PMC10532611

[10] O'Sullivan JDB, Bullen A, Mann ZF. Mitochondrial form and function in hair cells. Hear Res. 2023;428:108660. 36525891 10.1016/j.heares.2022.108660 PMC10227193

[11] Hibino H, Horio Y, Inanobe A, et al An ATP-dependent inwardly rectifying potassium channel, KAB-2 (Kir4.1), in cochlear stria vascularis of inner ear: Its specific subcellular localization and correlation with the formation of endocochlear potential. J Neurosci. 1997;17(12):4711–4721. 9169531 10.1523/JNEUROSCI.17-12-04711.1997 PMC6573344

[12] Van Camp G, Smith RJ. Maternally inherited hearing impairment. Clin Genet. 2000;57(6):409–414. 10905659 10.1034/j.1399-0004.2000.570601.x

[13] Zhang Y, Fu X, Li Y, et al Macrophage-mediated immune response aggravates hearing dysfunction caused by the disorder of mitochondrial dynamics in cochlear hair cells. Hum Mol Genet. 2023;32(7):1137–1151. 36331344 10.1093/hmg/ddac270

[14] Santarelli R, Rossi R, Scimemi P, et al OPA1-related auditory neuropathy: Site of lesion and outcome of cochlear implantation. Brain. 2015;138(Pt 3):563–576. 25564500 10.1093/brain/awu378 PMC4339771

[15] Santarelli R, La Morgia C, Valentino ML, et al Hearing dysfunction in a large family affected by dominant optic atrophy (OPA8-related DOA): A human model of hidden auditory neuropathy. Front Neurosci. 2019;13:501. 31191217 10.3389/fnins.2019.00501 PMC6546873

[16] Chinnery PF, Elliott C, Green GR, et al The spectrum of hearing loss due to mitochondrial DNA defects. Brain. 2000;123(Pt 1):82–92. 10611123 10.1093/brain/123.1.82

[17] Jonard L, Marlin S, Louha M, et al Molecular diagnosis of genetic deafness. Clin Biochem. 2011;44(7):510–511. 22036352 10.1016/j.clinbiochem.2011.02.031

[18] Shields C, Sladen M, Bruce IA, et al Exploring the correlations between measures of listening effort in adults and children: A systematic review with narrative synthesis. Trends Hear. 2023:27:23312165221137116. 36636020 10.1177/23312165221137116 PMC9982391

[19] Pizzamiglio C, Machado PM, Thomas RH, et al COVID-19-related outcomes in primary PMD: An international study. Neurology. 2022;98(14):576–582. 35190464 10.1212/WNL.0000000000200240 PMC8992603

[20] Male AJ, Koohi N, Holmes SL, Pitceathly RDS, Kaski D. Acceptability of audiovestibular assessment in the home: A patient survey. Audiol Res. 2024;14(3):545–553. 38920966 10.3390/audiolres14030045 PMC11200979

[21] Heinrich A, Henshaw H, Ferguson MA. The relationship of speech intelligibility with hearing sensitivity, cognition, and perceived hearing difficulties varies for different speech perception tests. Front Psychol. 2015;6:782. 26136699 10.3389/fpsyg.2015.00782 PMC4468362

[22] Strori D, Souza PE. The role of working memory in speech recognition by hearing-impaired older listeners: Does the task matter? Int J Audiol. 2023;62(11):1067–1075. 36285707 10.1080/14992027.2022.2128445 PMC10130232

[23] Gallun FJ, Diedesch AC, Kampel SD, Jakien KM. Independent impacts of age and hearing loss on spatial release in a complex auditory environment. Front Neurosci. 2013;7:252. 24391535 10.3389/fnins.2013.00252 PMC3870327

[24] Nasreddine ZS, Phillips NA, Bédirian V, et al The Montreal Cognitive Assessment, MoCA: A brief screening tool for mild cognitive impairment. J Am Geriatr Soc. 2005;53(4):695–699. 15817019 10.1111/j.1532-5415.2005.53221.x

[25] Schaefer AM, Phoenix C, Elson JL, McFarland R, Chinnery PF, Turnbull DM. Mitochondrial disease in adults: A scale to monitor progression and treatment. Neurology. 2006;66(12):1932–1934. 16801664 10.1212/01.wnl.0000219759.72195.41

[26] Griffiths TD, Blakemore S, Elliott C, Moore BC, Chinnery PF. Psychophysical evaluation of cochlear hair cell damage due to the A3243G mitochondrial DNA mutation. J Assoc Res Otolaryngol. 2001;2(2):172–179. 11550526 10.1007/s101620010061 PMC3201187

[27] Sakata A, Kashio A, Koyama H, et al Long-term progression and rapid decline in hearing loss in patients with a point mutation at nucleotide 3243 of the mitochondrial DNA. Life (Basel). 2022;12(4):543. 35455034 10.3390/life12040543 PMC9033132

[28] Kishimoto-Urata M, Urata S, Fujimoto C, Yamasoba T. Role of oxidative stress and antioxidants in acquired inner ear disorders. Antioxidants (Basel). 2022;11(8):1469. 36009187 10.3390/antiox11081469 PMC9405327

[29] Fujimoto C, Yamasoba T. Mitochondria-targeted antioxidants for treatment of hearing loss: A systematic review. Antioxidants (Basel). 2019;8(4):109. 31022870 10.3390/antiox8040109 PMC6523236

[30] Di Stadio A, Pegoraro V, Giaretta L, Dipietro L, Marozzo R, Angelini C. Hearing impairment in MELAS: New perspectives in clinical use of microRNA, a systematic review. Orphanet J Rare Dis. 2018;13(1):35. 29466997 10.1186/s13023-018-0770-1 PMC5822652

[31] Cameron S, Dillon H. Development and validation of the listening in spatialized noise-sentences test (LiSN-S). Ear Hear. 2011;32(3):363–377. 10.1097/AUD.0b013e318031267f 17496671

[32] Rance G, Starr A. Pathophysiological mechanisms and functional hearing consequences of auditory neuropathy. Brain. 2015;138(Pt 11):3141–3158. 26463676 10.1093/brain/awv270

[33] Przewoźny T, Gójska-Grymajło A, Szmuda T, Markiet K. Auditory spatial deficits in brainstem disorders. Neurol Neurochir Pol. 2015;49(6):401–411. 26652875 10.1016/j.pjnns.2015.10.001

[34] Yin TCT, Smith PH, Joris PX. Neural mechanisms of binaural processing in the auditory brainstem. Compr Physiol. 2019;9(4):1503–1575. 31688966 10.1002/cphy.c180036

[35] Brughera A, Mikiel-Hunter J, Dietz M, McAlpine D. Auditory brainstem models: Adapting cochlear nuclei improve spatial encoding by the medial superior olive in reverberation. J Assoc Res Otolaryngol. 2021;22(3):289–318. 33861395 10.1007/s10162-021-00797-0 PMC8110671

[36] Marozzo R, Pegoraro V, Dipietro L, Ralli M, Angelini C, Di Stadio A. Can miR-34a be suitable for monitoring sensorineural hearing loss in patients with mitochondrial disease? A case series. Int J Neurosci. 2020;130(12):1272–1277. 32079439 10.1080/00207454.2020.1731505

[37] Meyer C, Grenness C, Scarinci N, Hickson L. What is the International Classification of Functioning, Disability and Health and why is it relevant to audiology? Semin Hear. 2016;37(3):163–186. 27489397 10.1055/s-0036-1584412 PMC4954783

[38] Slade K, Plack CJ, Nuttall HE. The effects of age-related hearing loss on the brain and cognitive function. Trends Neurosci. 2020;43(10):810–821. 32826080 10.1016/j.tins.2020.07.005

[39] Kllmeier B, Kiessling J. Functionality of hearing aids: State-of-the-art and future model-based solutions. Int J Audiol. 2018;57(Suppl3):S3–S28. 10.1080/14992027.2016.1256504 27951738

[40] Kanemoto K, Kashio A, Ogata E, et al Cochlear implantation in patients with mitochondrial gene mutation: Decline in speech perception in retrospective long-term follow-up study. Life (Basel). 2022;12(4):482. 35454973 10.3390/life12040482 PMC9029697

