# Supplementary material for: Beyond the cochlea: exploring the multifaceted nature of hearing loss in primary mitochondrial diseases
Source: Brain Commun. 2024 Oct 24;6(6):fcae374. doi: 10.1093/braincomms/fcae374 (PMC11583428; doi:10.1093/braincomms/fcae374)
Supplement: fcae374_Supplementary_Data [file fcae374_supplementary_data.pdf]

Supplementary Material

MANCOVA PTA

In the assessment of auditory thresholds, the MANCOVA revealed significant differences across diagnostic groups. Specifically, for the average left PTA, the m.3243A>G/T group demonstrated elevated thresholds compared to the nuclear mutation variants group (Mean Difference = 29.182, 95% CI [12.859, 45.504], p < .001), the clinicopathological group (Mean Difference = 26.470, 95% CI [7.773, 45.167], p = .006), and other mtDNA mutation variants group (Mean Difference = 16.316, 95% CI [4.702, 27.930], p = .007).

Similarly, for the average right PTA, the m.3243A>G/T group showed higher thresholds than the nuclear mutation variants group (Mean Difference = 28.770, 95% CI [14.237, 43.302], p < .001), the clinicopathological group (Mean Difference = 22.624, 95% CI [5.977, 39.271], p = .009), and other mtDNA mutation variants group (Mean Difference = 15.049, 95% CI [4.708, 25.390], p = .005).

Raised thresholds were also observed in the high-frequency hearing assessments. For the left high-frequency average, significant differences were found when comparing the m.3243A>G/T group to the nuclear mutation group (Mean Difference = 36.509, 95% CI [18.107, 54.910], p < .001), the nuclear mutation excluding mt gene group (Mean Difference = 36.322, 95% CI [15.243, 57.401], p = .001), and other Mt DNA mutation group (Mean Difference = 21.816, 95% CI [8.722, 34.910], p = .001).

The right high-frequency average showed a similar pattern with the m.3243A>G/T group exhibiting greater thresholds compared to the nuclear mutation group (Mean Difference = 35.565, 95% CI [18.470, 52.661], p < .001), the clinicopathological group (Mean Difference =

34.081, 95% CI [14.498, 53.663], $p < .001$), and other mtDNA mutation group (Mean Difference = 20.605, 95% CI [8.440, 32.769], $p = .001$).

MULTINOMIAL LOGISTIC REGRESSION ANALYSIS

We aimed to determine the predictive role of genetic diagnosis in hearing impairment categories, with a focus on understanding the additional contributions of age and disease severity (as measured by NMDAS scores). We used a multinomial logistic regression approach, constructing a series of nested models to evaluate the impact of these variables.

The models were structured as follows: Model 1 included only "Genetic Groups," Model 2 added "Age," Model 3 included "Genetic Groups" and "NMDAS," and Model 4 combined all three variables. The models were compared using the Akaike Information Criterion (AIC) and Bayesian Information Criterion (BIC) to establish the most appropriate balance between model fit and complexity. The key findings from our nested model analysis are summarised below:

Model 1 (Only Genetic Groups) showed AIC and BIC values of 195.91 and 213.79, respectively. Model 2 (Genetic Groups + Age) revealed a slight improvement in AIC and BIC values to 194.10 and 220.91. Model 3 (Genetic Groups + NMDAS) presented the most significant improvement, yielding the lowest AIC and BIC values of 186.56 and 213.37, respectively. Model 4 (Genetic Groups + Age + NMDAS), while comprehensive, did not provide a justifiable improvement over Model 3, as indicated by higher AIC and BIC values of 190.50 and 226.24.

Based on these results, Model 3 was identified as the optimal model. This model, which includes "Genetic Groups" and "NMDAS," provided the best balance of explanatory power and parsimony. The findings underscore the significant role of specific genetic diagnoses in predicting hearing impairment categories, augmented by the influence of auditory function as

measured by NMDAS scores. The addition of "Age" as a covariate did not significantly enhance the predictive capacity of the model, suggesting that the effects of genetic factors and auditory function are relatively independent of age within the context of our study.

The results suggest a significant association of NMDAS scores with Hearing Categories 2 (neural/central), 4 (peripheral and neural/central), and 5 (inconclusive) for Genetic Group 1 (m.3243 A>G/T), indicating that individuals in this group have a higher likelihood of falling into these categories when compared to the reference category (other mtDNA mutation). In contrast, Genetic Groups 2 (nuclear mutation) and 3 (clinicopathological) showed no significant associations with these hearing categories, except for a mild association in Category 4 (peripheral and central).

Supplementary Figure 1: the figure presents the individual data points for the groups with fewer than 10 samples, specifically focusing on the Nuclear Gene Variant and Clinicopathological groups. Panels A display the Listening in Spatialized Noise-Sentences (LiSN-S), Pure Tone Audiometry (PTA) results for the right and left ears at various frequencies (250 Hz, 500 Hz, 1000 Hz, 2000 Hz, 4000 Hz, 8000 Hz), and Quick Speech in Noise (QSiN) for the left and right ear for the Nuclear Gene Variant group. Panel B displays the same metrics for the Clinicopathological group.

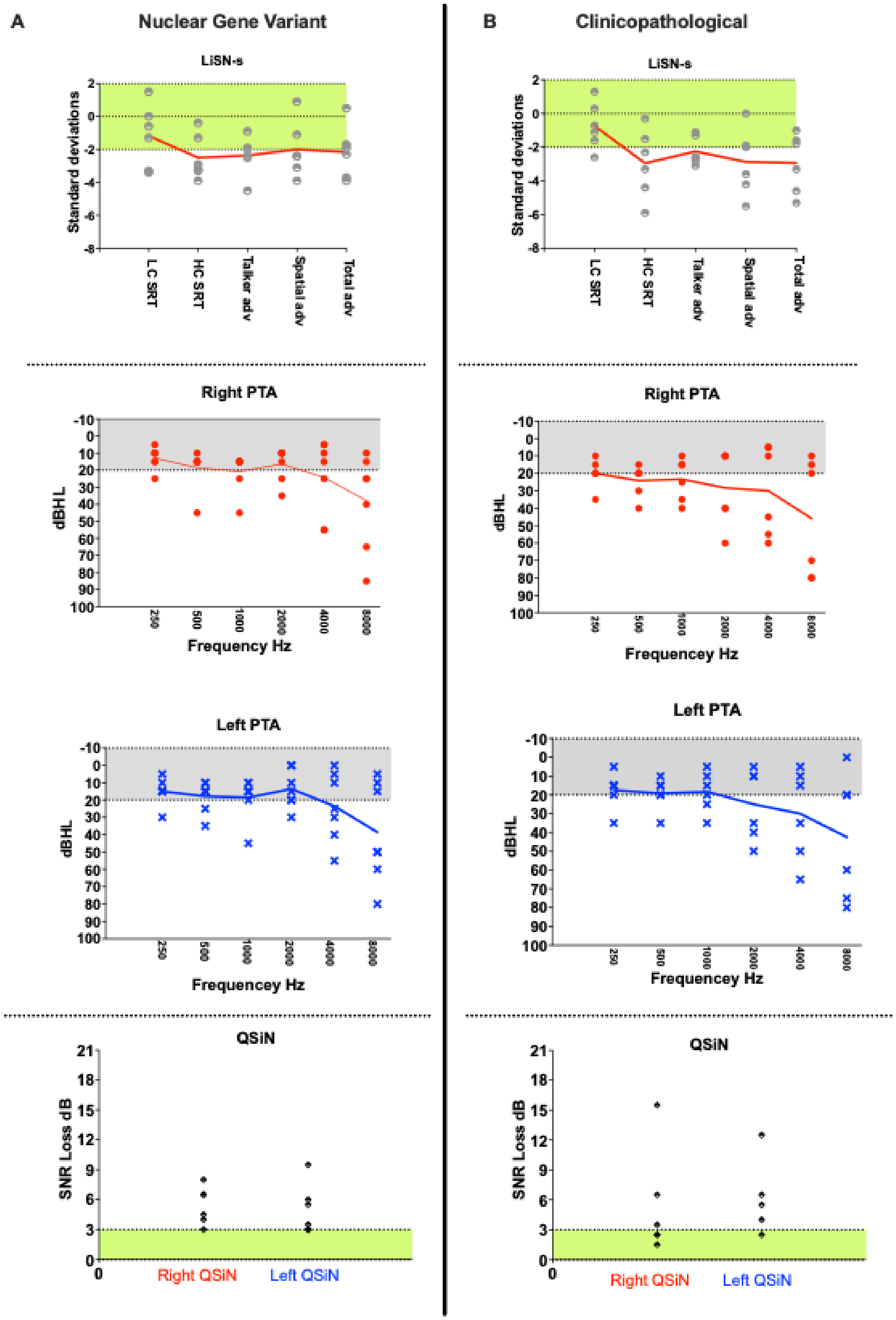

A
Nuclear Gene Variant
B
Clinicopathological
LiSN-s
Standard deviations
LC SRT
HC SRT
Talker adv
Spatial adv
Total adv
Right PTA
Left PTA
dBHL
Frequencey Hz
250
500
1000
2000
4000
8000
QSiN
SNR Loss dB
Right QSiN
Left QSiN

Supplementary Table 1: Demographic distribution of mitochondrial disease variants and associated clinical features as per the Newcastle Mitochondrial Disease Adult Scale assessment. KEY: NMDAS - Newcastle mitochondrial disease adult scale, mtDNA - mitochondrial DNA, MIDD - Maternally Inherited Diabetes and Deafness, MERRF - Myoclonic Epilepsy with Ragged Red Fibers, MELAS - Mitochondrial Encephalomyopathy, Lactic Acidosis, and Stroke-like episodes, SANDO - Sensory Ataxic Neuropathy, Dysarthria, and Ophthalmoparesis.

GENETIC GROUP	GENETIC DIAGNOSIS	NMDAS	PHENOTYPE								MUTANT LOAD (%)				MOCA (n/30) (Level of impairment: normal=26-30, mild=18-25, moderate=10-17, severe=<10)	4 Frequency average	
		Sum completed NMDAS questions(25)	MIDD	MERRF	MELAS	SANDO	Kearns-Sayre syndrome	Leigh syndrome	Asymptomatic	Other	Age-adjusted blood m.m.3243A>GA>G	Blood	Urine	Muscle		Left ear	Right ear
m.3243A>G	m.m.3243A>GA>G, *MT-TL1*	14								Y	38	8		78	22	58.75	56.25
m.3243A>G	m.m.3243A>GA>G, *MT-TL1*	3									28	5	28		26	48.75	76.25
m.3243A>G	m.m.3243A>GA>G, *MT-TL1*	22	Y								56	9			25	60	63.75

m.3243A>G	m.m.3243A>GA>G, *MT-TL1*	39			Y						70	23			23	42.5	40
m.3243A>G	m.m.3243A>GA>G, *MT-TL1*	33	Y							Y	60	14	48	80	25	102.5	103.75
m.3243A>G	m.m.3243A>GA>G, *MT-TL1*	11			Y						87	23	71	85	29	70	68.75
m.3243A>G	m.m.3243A>GA>G, *MT-TL1*	35								Y	34	12	31		15	53.75	56.25
m.3243A>G	m.m.3243A>GA>G, *MT-TL1*	21								Y	71	21	14		25	77.5	81.25
m.3243A>G*	m.m.3243A>GA>G, *MT-TL1*	17								Y					22		
m.3243A>G	m.m.3243A>GA>G, *MT-TL1*	11	Y								57	20	54		26	36.25	33.75
m.3243A>G	m.m.3243A>GA>G, *MT-TL1*	3									8	2	11		27	10	12.5
m.3243A>G	m.m.3243A>GA>G, *MT-TL1*	6											45		29	31.25	32.5
m.3243A>G	m.m.3243A>GA>G, *MT-TL1*	3	Y								96	22			22	57.5	87.5
m.3243A>G	m.m.3243A>GA>G, *MT-TL1*	5							Y		43	14			26	8.75	10
m.3243A>G	m.m.3243A>GA>G, *MT-TL1*	2									61	20			22	33.75	37.5
m.3243A>G	m.m.3243A>GA>G, *MT-TL1*	18	Y								65	17			30	55	55
m.3243A>G	m.m.3243A>GA>G, *MT-TL1*	6									43	10	62		29	27.5	28.75
m.3243A>G	m.m.3243A>GA>G, *MT-TL1*	25								Y	35	7	32		25	62.5	62.5
m.3243A>G	m.m.3243A>GA>G, *MT-TL1*	5									91	28			30	16.25	17.5
m.3243A>G	m.m.3243A>GA>G, *MT-TL1*	16	Y								48	9	40		9999	52.5	50
m.3243A>G	m.m.3243A>GA>G, *MT-TL1*	13	Y								32	9			9999	17.5	20
m.3243A>G	m.m.3243A>GA>G, *MT-TL1*	23	Y								35	5			14	67.5	58.75

m.3243A>G	m.m.3243A>GA>G,<br>*MT-TL1*	15	Y								11	3			9999	17.5	16.25
m.3243A>G*	m.m.3243A>GA>G,<br>*MT-TL1*	18	Y								100	37			23		
m.3243A>G	m.m.3243A>GA>G,<br>*MT-TL1*	15	Y							Y	73	19			27	42.5	45
m.3243A>G	m.m.3243A>GA>G,<br>*MT-TL1*	12	Y								57	14			26	32.5	31.25
m.3243A>G	m.m.3243A>GA>G,<br>*MT-TL1*	2							Y		100	59			15	12.5	7.5
m.3243A>G	m.m.3243A>GA>G,<br>*MT-TL1*	15								Y			52		20	16.25	17.5
m.3243A>G	m.m.3243A>GA>G,<br>*MT-TL1*	3							Y		67	29			26	5	10
m.3243A>G	m.m.3243A>GA>G,<br>*MT-TL1*	5								Y	69	31	71		30	13.75	10
m.3243A>G	m.m.3243A>GA>G,<br>*MT-TL1*	5									50	17	48		20	97.5	107.5
m.3243A>G	m.m.3243A>GA>G,<br>*MT-TL1*	13								Y	91	38			22	40	32.5
m.3243A>G	m.m.3243A>GA>G,<br>*MT-TL1*	0							Y		80	31	51		23	13.75	20
m.3243A>G	m.m.3243A>GA>G,<br>*MT-TL1*	9									51	17			29	47.5	48.75
m.3243A>G	m.m.3243A>GA>G,<br>*MT-TL1*	15	Y								73	11			15	81.25	73.75
m.3243A>G	m.m.3243A>GA>G,<br>*MT-TL1*	10									77		46		27	12.5	12.5
m.3243A>G	m.m.3243A>GA>G,<br>*MT-TL1*	15	Y								100	31	34		27	43.75	27.5
m.3243A>G	m.m.3243A>GA>G,<br>*MT-TL1*	3								Y	100	66	73		9999	16.25	15
m.3243A>G	m.m.3243A>GA>G,<br>*MT-TL1*	20	Y								48	10			30	41.25	37.5
m.3243A>G	m.m.3243A>GA>G,<br>*MT-TL1*	4									4	2			27	3.75	1.25
m.3243A>G	m.m.3243A>GA>G,<br>*MT-TL1*	7												59	29	25	23.75

m.3243A>G	m.m.3243A>GA>G, *MT-TL1*	10								Y		62			25	61.25	66.25
m.3243A>GA>T	m.m.3243A>GA>T, *MT-TL1*	17								Y				75	29	8.75	10
Other mtDNA pathogenic variants	m.8344A>G, *MT-TK*	19		Y									70		25	18.75	22.5
Other mtDNA pathogenic variants	m.8344A>G, *MT-TK*	7		Y								53			23	22.5	21.25
Other mtDNA pathogenic variants	m.8344A>G, *MT-TK*	27		Y								86			23	43.75	45
Other mtDNA pathogenic variants	m.8344A>G, *MT-TK*	3		Y						Y		58			26	17.5	16.25
Other mtDNA pathogenic variants	m.8344A>G, *MT-TK*	21		Y								79			19	48.75	37.5
Other mtDNA pathogenic variants	m.10158t>c, *MT-ND3*	12										2	38	39	26	17.5	21.25
Other mtDNA pathogenic variants	m.12258A>G, *MT-TS2*	13											78	30	23	55	56.25
Other mtDNA pathogenic variants	m.13513G>A, *MT-ND5*	5										2	19		26	16.25	13.75
Other mtDNA pathogenic variantss	m.14674T>C, *MT-TE*	26								Y				100	11	41.25	36.25
Other mtDNA pathogenic variants	m.4300A>G, *MT-TI*	0							Y			100			9999	6.25	6.25
Other mtDNA pathogenic variantss	m.10038G>A, *MT-TG*	14										15	40	92	25	71.25	75
Other mtDNA pathogenic variants	mtDNA rearrangement	10					Y								26	8.75	5
Other mtDNA pathogenic variants	Single mtDNA deletion	9													19	17.5	20
Other mtDNA pathogenic variants	Single mtDNA deletion	8													25	13.75	12.5
Other mtDNA pathogenic variants	Single mtDNA deletion	10								Y					20	15	15
Other mtDNA pathogenic variants	Single mtDNA deletion	8													18	12.5	12.5
Other mtDNA pathogenic variants	Single mtDNA deletion	14								Y					27	22.5	17.5

Other mtDNA pathogenic variants	Single mtDNA deletion	13													15	15	17.5
Clinicopathological	Multiple mtDNA deletions	9													23	30	18.75
Nuclear mutation	AD POLG	37				Y									26	30	32.5
Nuclear mutation	AD POLG	6													20	11.25	6.25
Nuclear mutation	AD OPA1	14													27	27.5	22.5
Nuclear mutation	AD PEO1	20								Y					27	16.25	18.75
Nuclear mutation	AR RNASEH1	28								Y					25	13.75	20
Nuclear mutation	AR SURF1	9						Y							27	11.25	10
Clinicopathological diagnosis	Multiple mtDNA deletions (nuclear maintenance gene panel negative)	18													27	41.25	36.25
Clinicopathological diagnosis	Clinicopathological diagnosis – no genetic confirmation	14								Y					29	13.75	7.5
Clinicopathological diagnosis	Clinicopathological diagnosis – no genetic confirmation	10								Y					25	12.5	15
Clinicopathological diagnosis	Clinicopathological diagnosis – no genetic confirmation	23													9999	33.75	31.25
Clinicopathological diagnosis	Clinicopathological diagnosis – no genetic confirmation	25													19	46.25	37.5
Clinicopathological diagnosis	Clinicopathological diagnosis – no genetic confirmation	15													26	11.25	11.25
